# Supplementary material for: Are carbon emissions trading and green financial instruments synergistic? -Comprehensive quantitative research based on content analysis
Source: PLoS One. 2024 Mar 7;19(3):e0298601. doi: 10.1371/journal.pone.0298601 (PMC10919601; doi:10.1371/journal.pone.0298601)
Supplement: S1 Data — (ZIP) [file pone.0298601.s001.zip › Data/地级市Z_F工作报告.zip!/╜¡╬≈/╝¬░▓╩╨/└┤╫╘╣1⁄2╓┌║┼í╛┬φ┐╦╩2╛▌═°í┐_2014.docx]

各位代表：

　　现在，我代表市人民政府向大会报告工作，请予审议，并请各位市政协委员和列席会议的同志提出意见。

　　一、2013年工作回顾

　　2013年是深入贯彻落实党的十八大精神的开局之年。在省委、省政府和市委的坚强领导下，全市上下牢牢抓住中央苏区振兴发展、吉泰走廊战略升级等重大历史机遇，迎难而上，奋发有为，扎实苦干，较好完成了市三届人大三次会议确定的主要目标任务，“发展升级、小康提速、绿色崛起、实干兴吉”迈出了新步伐，建设开放繁荣秀美幸福新吉安出现了新局面。

　　———经济发展逆势上扬，升级提速的态势进一步显现。全市生产总值1120亿元、增长11%，财政总收入169亿元、增长18.5%，以及规模工业增加值、实际利用外资、引进内资、外贸出口、贷款余额、农民人均纯收入、限额以上社会消费品零售总额等9项指标增幅居全省前列，11项指标增幅在全省排位前移，缩小差距、进位赶超取得佳绩，成长性明显增强。城乡居民收入与经济同步增长，财政收入占GDP、税收占财政收入比重分别提高0.9和3.4个百分点，发展质量与居民收入同步提升，协同性明显增强。三次产业齐头并进，投资、消费、出口“三驾马车”共同发力，均衡性明显增强。工业用电量增长15.5%，商品房销售面积增长51.3%，客货运周转量、金融机构存贷款、企业景气指数等其他风向性指标也都持续上扬，匹配性明显增强，有力佐证了吉安经济的活跃与向好。

　　———各项事业统筹推进，发展的全面性、协调性进一步增强。在加快经济建设的同时，坚持负责任发展、集约集优发展、统筹发展和绿色发展，大力推进民生工程、社会事业和生态建设。教育卫生事业进入大投入大建设大提升时期，社会保障体系不断完善。文化建设大发展、大繁荣，传承优秀地域文化的自信自觉更加坚定，政府重文化、社会办文化、企业经营文化形成气候，各类文艺创作和文化作品赢得各方好评。绿色发展理念深入人心，“美丽中国”在吉安得到生动实践，城乡建设进一步彰显绿色生态文化内涵和地域特色。吉安喜获全国绿化模范城市、全省“森林城乡、绿色通道”优秀设区市、全省文明城市、全省社会管理综合治理优秀市。公众安全感指数在全省保持前位。

　　———改革创新破难前行，解放思想、大胆实践的局面进一步形成。把解放思想、改革创新作为发展原动力，找准制约发展的突出问题和羁绊，在重点领域、关键环节寻求突破。对县域经济的“松绑给力”，有力助推了竞相发展；工业园区的机制创新，提升了自主发展能力；城市建设的体制创新，大大增添了开发建设的生机活力；社会事业管理模式的探索实践，成功推进了文化艺术中心托管于保利集团、吉安职业技术学院与深圳高职院合作办学、城南医院托管于上海东方医院，有力促进了职业教育和医疗卫生站在高起点上。省委十三届七次全会后，进一步在解放思想中统一认识，深入广泛调研，形成了六个升级提速的行动计划，重点在吉泰走廊、县域经济、电子信息产业、红色旅游精品城市等方面探索新举措。特别是党的十八届三中全会后，在市委统一组织制定的全面深化改革“1+12”体系中，研究启动先行先试、转变政府职能、稳步推进改革的“试、转、稳”改革措施，谋求改革发展新优势。

　　一年来，我们重点抓了以下工作：

　　（一）突出重点战略带动，区域经济快速发展。坚定不移地昂起龙头、挺起脊梁、立起支点，着力打造支撑吉安进位赶超的重点板块。井开区东区拓展、西区提升加快推进。东区路网构架基本形成，项目落户初见成效；西区综合物流中心投入使用，出口加工区封关运行，电子专业市场主体完工，井冈山卷烟厂异地改造项目顺利启动，航盛、柏兆、合力泰等一批项目相继投产。井开区规模工业主营业务收入400亿元、增长30%，完成税收10.8亿元、增长95.6%。吉泰走廊跻身全省新一轮战略布局，在“加码、加压、加力、加速”中发力。电子信息、医药化工、先进装备制造等主导产业加快集聚，对全市的支撑和引领作用进一步显现。走廊实现生产总值561亿元，占全市的50.1%，提高1.5个百分点；财政总收入占全市的54.3%，提高1个百分点；规模工业增加值占全市的55.6%，提高1.8个百分点。“一核两翼三带”城镇群体系加速形成，井开区、吉安县、吉水县融城步伐进一步加快，10个优先发展重点镇及功能区建设深入推进，公共交通贯穿南北70公里，天然气管网正向吉水、泰和县城延伸，统一的劳动力市场初步形成，产城互动、城乡一体的发展格局更加明显。县域经济加快成长，差异化竞相发展格局进一步展现。制定出台了扶持县域经济加快发展的政策措施，推动县域打造主导产业，发展优势产业，培育本土产业，各县市区产业的规模、特色和集聚度进一步提升。新干的盐化、箱包皮具、玻璃灯饰，永丰的碳酸钙，吉水的林化香料，万安的电子线路板，遂川的绿色食品，安福的液压机电，永新的皮制品等特色产业渐成规模。全市8个县区财政总收入过10亿元，其中泰和县迈上15亿元，吉安县迈上20亿元。

　　（二）坚持工业化核心战略，提质提速态势强劲。大力实施工业强攻，推进传统产业和战略性新兴产业融合发展取得新成效。全市规模工业增加值突破500亿元、增长14%，主营业务收入2400亿元、增长20%，工业对经济增长的贡献率55.3%。产业结构进一步优化，工业经济效益综合指数达到410%、提高5.8个百分点，企业实现利税260亿元、增长20%。战略性新兴产业加速集聚。主营业务收入突破千亿、达到1196亿元，占全市规模工业的49.8%，提高0.8个百分点。其中，电子信息产业突破400亿元，占全省比重提高2个百分点、达到43%。传统产业加快提升。加快振兴酒业、提振烟叶、提升茶叶、做大食品加工业，金佳谷物新厂区正式开建，堆花酒厂异地改造步伐加快，燕京啤酒退城进园扎实推进，遂川茶博园开工建设，狗牯脑茶等传统产业焕发勃勃生机。食品加工业主营业务收入215亿元，增长25%。工业园区快速推进。建成标准厂房84万平方米、公租房8300多套。园区项目攻坚年活动成效显著，实现主营业务收入2200亿元、增长21%，吉安县、泰和县、新干县、永丰县4个园区突破200亿元。

　　（三）坚持“双轮”驱动，服务业繁荣发展。遵循“主导产业抓集聚、重点产业抓规模、新兴产业抓培育”，多点发力，推进服务业加快发展，完成增加值342亿元、增长10.2%，实现地方税收57亿元、增长42.5%。商贸物流业加快发展。中心城区十大市场体系建设加速推进，沃尔玛等高端商场的开业明显提升了商业品质，吉安商业开始摆脱长期低端、发育不全的窘境。全市社会消费品零售总额突破300亿元，增长14%。“一中心、一平台、多园区”物流体系初步形成，物流平台与信息网络不断完善，全市货运周转量348亿吨公里，增长20%。旅游业持续发展。科学、有序、适度开发的理念进一步确立，围绕打造全国红色旅游精品城市，旅游业“三年大决战”首战打响，井冈山成功创建首批国家生态旅游示范区，武功山箕峰和杨思慕景区建设取得重大进展，青原山整体开发步伐加快，吉州窑遗址公园建成开园、千年龙窑成功复烧，中心城区及周边景区套票联动运行，“三山一江”战略和红色旅游一体化有了新格局。全市接待国内外游客2526万人次、增长20%，旅游总收入突破200亿元、增长21.4%；井冈山机场旅客吞吐量45万人次，增长12.5%。金融业加速发展。既有力保障了经济建设，又成为重要税收来源。全市金融机构各项贷款余额达717.8亿元、增长25.9%，金融业完成地方税收6.9亿元、增长30.3%，南昌银行、交通银行入驻吉安，村镇银行、小额贷款公司等金融机构不断涌现，市信贷担保公司成功组建。总部经济正在形成，庐陵新区10个总部大楼已规划到位并陆续开工建设。文化、健康、养老等新兴服务业加快发展。

　　（四）坚持以现代理念和百姓情怀抓农业，农村经济增产增效。在稳步发展粮食生产的基础上，大力调优农业结构，发展富民产业。粮食生产再获丰收。总产达到403万吨，）实现“十连丰”。特色富民产业方兴未艾。井冈蜜柚深受群众欢迎，落实种植面积15万亩，“老乡工程”落实农户8.7万户。同时新增高产油茶10万亩、花卉苗木5万亩、珍贵楠木8万亩、绿色蔬菜2.6万亩、茶叶3万亩，肉类、家禽、特种水产等大宗农产品产量均稳步增长。现代农业加快发展。推进规模化经营，新增土地流转面积36.5万亩、农民合作社845个，永丰等地家庭农场蓬勃兴起。永新县寿光蔬菜等一批农业招商项目已显成效，全市新增市级以上龙头企业57家，越来越多的社会资本参与现代农业开发。井冈山农业科技园建设迈出坚实步伐，县市区农业科技园全面启动，农产品精深加工取得新进展。以“六大扶贫工程”为重点的精准扶贫大力推进。全市实施整村推进扶贫项目3129个，完成扶贫移民搬迁1万多人，减少农村贫困人口11万人。峡江水利枢纽2.5万移民春节前可全部乔迁新居。农业发展基础不断夯实。333座小（二）型病险水库除险加固，13个小农水重点县年度任务全面完成。建设高标准农田16.4万亩，恢复和改善灌溉面积33万亩。完成195个乡镇基层农技站能力建设，落实农机购置补贴9500万元，农业服务体系更加健全。

　　（五）坚定不移扩大开放，发展活力进一步增强。紧紧铆住对外开放，久久为功，大力推进工业、城市建设、现代农业、现代服务业、社会事业全领域、全方位开放。突出招大引强，主抓对台招商，强化央企对接。全市引进亿元以上项目181个，引进内资375亿元、增长21.3%，实际利用外资6.8亿美元、增长21.1%。打造江西的台商集聚地再结硕果，引进台资项目40个，合同引资13.3亿美元，尤其是台湾志嘉艺术园、泰鑫文化园，不但带来投资，而且引入了新的建设理念。中国电科、中电投、华润集团等一批央企项目签约落户。坚持招商引资与引进订单、拓展市场相结合。新增生产型出口企业36家，全市完成出口总额33.2亿美元、增长29.2%。坚持对外开放与对内开放并举。鼓励扶持全民创业，新增私营企业4306户、个体工商户2.1万户，非公有投资完成790亿元，占全市固定资产投资的72.5%，提高1.6个百分点。积极争取重大政策。中央苏区振兴政策对接有力，获得民政部、住建部等12个部委对口支援帮扶。国家印刷产业包装基地、国家旅游扶贫试验区、国家电子信息高新技术产业化基地成功获批，吉安县工业园区升格省高新技术产业园。全市争取各类资金162.5亿元，增长12.1%，再创历史新高。重大项目建设取得突破。抚吉高速、井睦高速建成通车，衡茶吉铁路已经贯通，吉安东西出省快速通道正在形成，区位优势进一步提升。峡江水利枢纽工程2台机组并网发电；赣江石虎塘航电枢纽全面竣工；西气东输等项目取得重大进展，新增天然气管道153公里；输变电站建设投资8.28亿元，农网投资占全省六分之一，为全省之最，能源支撑更加有力。全市固定资产投资1090亿元，增长23%。

　　（六）加快推进城乡一体化，“文化庐陵、山水吉安”更具魅力和品相。坚持城乡建设同步推进、加快城市建设和提高城镇化水平同步推进，城乡面貌继续发生深刻变化。新增城镇建成区面积25平方公里，城镇化率提高2个百分点，达到43.6%。注重规划先行，抓好吉泰城镇群规划和城市总规修编，城区控规和小城镇、行政村规划覆盖率100%，30户以上自然村规划覆盖率95%，农村违法用地、违法建设行为得到有效遏制。注重城乡一体，推进城乡统筹、城乡同治。中心城区四座大桥、六条道路、五大组团加快建设。永和连心大桥、新井冈山大桥、庐陵大桥、吉水赣江大桥同时布局推进。吉州大道南延、阳明路西延、吉福路西延、城南专业市场大道，特别是105国道改道和金樟大道加快建设，打通中心城区外环通道，连通走廊核心区5个园区和沿线5个乡镇。庐陵新区步入发展“快车道”，滨江新区、城南新区、赣江西堤片区正在兴起；赋予吉州、青原两区自主开发的板块加快推进，金樟组团路网建设全面展开，青原山组团建设已经启动，井开区和敦厚组团大步融城，大吉安组团式发展格局渐入佳境。美丽乡村建设坚持全域规划、带状推进，镇村联动、镇为核心，绿化同步、产业配套，全年新建镇村联动点86个、乡村点1070个，吉安县敦永线、青原青东线、吉水峡江105线、泰和苏马线、万安韶高线、井冈山茨下线、新干沂大线、遂川遂井线等一批新的精品示范带加快形成。很多乡镇发生脱胎换骨的变化，14个乡镇成为全省百强中心镇。注重建管并重，“四城同创”取得阶段性成果，省级文明城市成功创建，省级卫生城市通过复审。大力推广创建“人文社区、温馨家园”，社区建设闯出新天地。县市城区旧城改造、新城建设步伐加快，推进了一批高品质的公共服务设施，功能与品位正在提升，吉水、新干、安福等县城和井冈山新城实现跨越式发展，吉安县成功创建国家卫生县城。注重绿色生态，重视山体、水面、湿地等生态系统的保护，在中心城区着力打造大尺度景观、大面积水域、大生态绿化，古后河绿廊、螺湖湾湿地公园成为金腰带、绿景线。美丽乡村建设坚持“八不八多”原则，保护了庐陵农村的大生态和田园风光。注重地域文化，坚持文化自信自觉，注重历史文化内涵和精神力量的传承。按照修旧如旧、建新如旧的原则，加快推进白鹭洲书院修缮、儒林里特色古街改造建设，城市文化底蕴得以保全。加强古村落保护，涌现出一大批彰显庐陵风格的好建筑、新镇村，18个镇村被评为全省历史文化名镇名村，9个古村被评为第一批中国传统村落，总数为全省之最。

　　（七）不断强化民生保障，群众生活进一步改善。始终坚持民本理念，践行民生优先，努力让发展成果惠及更多百姓。全市各级财政民生支出225亿元、比上年增加35亿元，76项民生实事全面完成。群众生活水平进一步提高。城镇居民人均可支配收入22348元，增长11%。农民人均纯收入8020元，增长13%。全市居民人均储蓄存款19985元，增长17.4%。物价保持基本稳定，居民消费价格总水平涨幅控制在2.3%。就业形势稳中向好，新增城镇就业5.6万人、转移农村劳动力8.4万人。社会保障体系不断完善，建立了城镇居民大病保险制度，事业单位工伤保险在全省率先实现全覆盖。福利院、光荣院、敬老院“三院”建设稳步推进，城乡社会救助水平进一步提高。居住环境继续改善，开工建设保障性住房1.63万套，基本建成1.31万套；以前所未有的力度推进棚户区改造，20个改造项目全面开工；全市完成无物业管理住宅小区改造项目95个，其中中心城区完成65个。新一轮公租房摇号选房顺利完成，中心城区2800户符合条件的家庭获得新居。农村危房改造完成1.6万户。生态建养卓有成效。"森林城乡、绿色通道"工程扎实推进。新增9个国家级生态乡镇、11个省级生态乡镇、14个省级生态村，井开区、吉州工业园被列为省级生态工业园。净空、净水、净土行动取得实效，节能减排目标全面完成。

　　（八）统筹推进各项事业，经济社会协调发展。社会事业投入持续加大，仅中心城区正在实施的教育、文化、卫生项目达19个，总投资49.6亿元。努力办人民满意的教育。中心城区重点布局建设了一批从学前教育到高等教育的项目，白鹭洲中学新校区、市特殊教育学校竣工投入使用，吉安职业技术学院、吉安一中新校区等一批重点项目加快推进，将原市委机关大院划给吉师附小改善办学条件。主动倾听和回应群众呼声，开展农村教育大调研，恢复农村教学点19个，变“孩子走读为老师走教”。全市完成农村义务教育薄弱学校改造项目304个、公办幼儿园建设359个。全市高考再创佳绩。设立圆梦助学金，全市崇文重教、济困助学蔚然成风。科技创新能力增强。全市专利授权量增长48.1%，开发省重点新产品59项，新建8个省级创新平台，博士后工作站实现零的突破。文体事业不断繁荣。低成本、本土化的文化精品大戏《记忆庐陵》入选江西省文艺创作繁荣工程，公演30多场，亮相京城，走出国门，广受好评。青原山净居寺被列为全国宗教界爱国主义教育基地。全民健身运动不断深入，第四届市运会成功举办。卫生事业不断发展。大力开展乡村医生签约服务和“家庭医生进社区”活动，选派医务人员到卫生室空白村蹲点服务。人口计生工作扎实推进，低生育水平持续稳定。创新和加强社会管理。深入推进社区网格化管理，切实加强网络舆情管理，基层基础群防群治进一步落实。食品药品安全和生产安全形势总体平稳。社会管理综合治理和信访维稳扎实有效，社会保持和谐稳定。驻吉部队和民兵预备役人员积极支持地方经济社会建设。国家安全、国防动员、拥军优属、人民防空等工作进一步加强，能源、交通、通信、商务等领域中央和省属单位服务地方经济社会发展积极作为、成效显著，各项事业有了新的发展。

　　过去的一年，我们进一步加强政府自身建设。扎实推进依法行政，自觉接受市人大的法律监督和市政协的民主监督，落实向市人大报告工作、向市政协通报工作制度，认真听取人大代表和政协委员的意见建议，全年办理市人大代表建议145件、市政协提案169件，办结率100%，满意和基本满意率100%。开展政府绩效管理，不断提升行政效能，建立“马上就办、马上就查”工作体系。推进简政放权，精简下放行政审批和公共服务322项，精简37.8%。严格遵守中央“八项规定：和反对“四风”要求，全面落实廉政建设责任制，加强行政权力运行监督，加大项目投资评审力度，市级项目评审节约资金6.1亿元。全面停止新建政府性楼堂馆所，全市“三公”经费支出下降7.7%。加强政府内部审计监察，强化资金监管，开展了民生资金管理使用情况监督检查等专项整治。进一步改进工作作风，深入开展“三进三解三促”活动，认真办好行风热线，受理办复群众来信来访来电2.2万件次，努力为人民群众排忧解难。

　　各位代表，过去的一年，我们在推进四化同步互动、三产共生演进和全面建成小康社会进程中迈出了坚实而可喜的步伐。成绩的取得，是省委、省政府和市委正确领导、科学决策的结果，是市人大、市政协及社会各界有效监督、大力支持的结果，是全市人民团结拼搏、奋发努力的结果。在此，我代表市人民政府，向奋斗在各条战线的全市广大干部群众，向历任老领导、离退休老同志，向各民主党派、工商联、无党派人士和社会团体，向驻吉人民解放军、武警官兵和公安消防干警，向中央和省驻吉单位，向所有关心、支持和参与吉安发展的同志们、朋友们和社会各界人士，表示衷心的感谢并致以崇高的敬意！

　　回顾过去一年，我们也清醒认识到当前经济社会发展还存在不少困难和问题：经济发展总体水平仍然较低，综合经济实力仍然不强，结构不优、总量不大。实体经济面临的困难依然较多，生产成本上升，要素保障受限。结构性就业矛盾突出，城乡居民增收难度大。科技创新能力不足，缺乏核心竞争力。体制机制障碍需要进一步突破，内生动力依然不强。我们的工作中，还存在诸多问题和不足。有些部门管理不严、工作不力，有些干部作风漂浮、工作不实，不作为、慢作为、乱作为现象仍时有发生，形式主义、官僚主义、享乐主义和奢靡之风等“四风”问题还不同程度地存在，政务环境还有待进一步改善。对此，我们一定高度重视，并认真加以解决。

　　二、2014年工作任务

　　2014年是深入贯彻落实党的十八届三中全会精神、全面深化改革的第一年，也是加快全面建成小康社会进程的关键之年。我们虽然面临不少挑战，但机遇大于挑战、有利条件更多。虽然国际经济形势依然错综复杂，但缓慢复苏的态势仍将延续，我们仍处于可以大有作为的重要战略机遇期。虽然国内经济存在下行压力，但长期向好的基本面没有改变。中央继续实施积极的财政政策和稳健的货币政策，特别是对全面深化改革作出了总体部署，为我们先行先试、突破体制机制障碍、激发发展动力活力提供了重大机遇。当前，我市加快发展的良好态势，为下步发展奠定了坚实基础;全市上下风清气正、团结奋进的良好政治生态，为我们加快发展提供了重要保障；广大干部群众昂扬向上、奋发进取的精神状态，积蓄了干事创业、共奔小康的蓬勃正能量。只要我们坚定信心、抢抓机遇，攻坚克难、开拓进取，就一定能推动全市经济社会发展迈出更大步伐。

　　2014年政府工作的总体要求是：全面贯彻落实党的十八届三中全会、中央经济工作会议和省委十三届八次全会、市委三届七次全会精神，坚定不移地推进“四化”同步互动、“三产”共生演进，改革创新、扩大开放，提速升级、稳中求进，努力保障和改善民生、促进社会和谐，迈开“发展升级、小康提速、绿色崛起、实干兴吉”新步伐，为建设开放繁荣秀美幸福新吉安奠定坚实基础。今年全市经济社会发展主要预期目标是：生产总值增长11%，规模工业增加值增长14%，财政总收入增长15%，固定资产投资增长22%，社会消费品零售总额增长14%；引进省外资金增长22%，实际利用外资增长15%，外贸出口增长12%；城镇居民人均可支配收入增长12%，农民人均纯收入增长13%；居民消费价格总水平涨幅控制在3.5%左右；人口自然增长率控制在7.8‰以内；单位生产总值能耗下降3.2%。

　　在实施过程中，我们将以改革创新为主动力，以扩大开放为主战略，以提速升级为总目标，以稳中求进为总基调，推动经济社会发展稳步上扬，保持发展速度高于全国、全省平均水平，在提高质量效益中做大经济总量，在调优经济结构中推动发展升级，在加快发展中让群众得到更多实惠。

　　围绕上述目标，重点抓好以下十个方面的工作：

　　（一）全面深化改革，充分激发经济发展动力活力。坚持问题导向、发展导向、市场导向和民生导向，加紧落实市委全面深化改革“1+12”意见。突出经济体制改革主轴牵引，突出吉泰走廊先行先试。先行启动中央已明确、吉安已看准、群众最期盼，且属于地方事权的30项改革，尤其加快推进已形成实施意见的12项改革，探索跟进涉及面广、情况复杂且必须推进的20项改革，最大限度释放改革红利。

　　推进现代市场体系建设。按照“非禁即入”原则，实行宽进政策，制定负面清单，消除各种障碍和壁垒，推动非公有制经济加快发展。积极发展混合所有制经济，鼓励非国有资本参股政府新投资项目。积极推进工商登记制度改革，推行“先照后证”、注册资本“认缴登记制”，“年检制”为“年度报告制”。进一步开放公共服务领域，引导和支持民间资本投向市政、交通等公用事业，以及教育、医疗、养老、健康等服务业。积极探索政府购买公共服务新模式。

　　加快转变政府职能。着力解决“越位、缺位、错位”问题，把简政放权、提高效能、优化服务与政府机构改革有机结合起来，在三季度前完成市县两级改革任务。精简下放审批权，做到能放则放、应放尽放，变事前审批为事中事后监管；整合下沉执法权，推动执法权重心下移，变多级执法为综合执法；优化放开办事权，推进事业单位分类改革和去行政化，引入竞争，优化服务。进一步提高行政效能，健全“马上就办、马上就查”工作体系；实施流程再造，积极探索“委托协作、合并主办”机制；提高电子政务水平，实行窗口服务、网上审批。

　　深化财税金融体制改革。加快推进市县部门预算改革，建立健全规范统一、公开透明的部门预算体系。扎实推进“营改增”工作。有序开展政府融资，保障项目资金接续能力。控制融资成本，健全预警和偿还机制，有效防范政府债务风险。加强政府投资项目资金监管，确保项目资金安全。大力引进培育金融机构，推进符合条件的小额贷款公司改制为村镇银行，鼓励各类资本依法设立或参与组建民营银行。开展民间借贷登记服务中心试点，筹建资产管理公司和私募基金。推进中小企业直接债务融资，加快发行吉泰走廊区域集优集合票据，扶持企业上市。筹建产业基金，扶持潜力企业。

　　激发基层发展活力。推进扩权强镇，选择24个重点镇进行试点，赋予财政收支权、行政管理权和必要的执法权，理顺站所管理体制，下达专项用地指标，增强资金安排自主权，提高乡镇产业发展与公共服务能力。推进工业园区扩权提效，赋予园区更大的财政自主权、项目运作权和行政管理权，健全园区高效管理体制和封闭运行机制，激发园区自我发展能力。

　　统筹推进其它各项改革。围绕“五位一体”布局，着力推进文化、社会、生态等领域改革。深化文化体制改革，坚持市场导向，创新管理体制和运行机制，建立公共文化服务体系和文化产业体系。深化教育领域综合改革，创新农村师资管理、待遇激励、职称评定等新机制；探索职业教育新路径，推进职业教育资源整合，加快打造品牌职校和职业教育集聚区。深化生态体制改革，加快建立市立公园、生态红线管控制度。突出经济发展和民生诉求，统筹推进人才培养引进、创新创业就业、农业经营机制、旅游开发经营、农村医疗卫生、社会保障、户籍制度等各领域改革。

　　（二）聚焦聚集吉泰走廊，谋求区域发展新成效。推进区域发展体制机制改革创新，加强与中央和省对口支援对接，用好重大政策机遇，进一步形成重点战略引领、各个区域协调发展的格局。

　　加快吉泰走廊建设。推进吉泰走廊先行先试，加快产业布局、基础设施、公共服务、社会保障、生态保护、招商政策和要素市场等一体化进程，提升整体优势和集聚效应，力争生产总值增长13%，规模工业增加值增长18%，城镇化率提高3个百分点、达到50.2%。合理划分三县四区产业分工，实现优势互补、错位发展。加快“一核两翼三带”吉泰城镇群建设和产城融合步伐，以中心城区为引领，加快井开区、吉安县和吉水县融城步伐，打破重点镇行政区划，推进走廊城镇组团式发展。逐步统一走廊区域各园区用地、水电气价格，加快建立统一的人力资源市场、专业市场、金融市场。实施公交优先、公交优惠、公交优质“三优”工程，实现公交由国道向省县道延伸拓展。加快推进吉泰走廊公共垃圾发电、天然气管网延伸等项目。统筹制定走廊区域招商引资、产业发展政策措施，建立区域内利益共享机制。

　　提升井开区发展水平。加快推进东区开发和西区提升步伐。东区完成基础设施主框架，深圳大桥上半年全面建成，科技园投入使用，天际光电、丰年精密、威力能源等一批电子信息、先进制造、新能源等重大项目投产见效。加快推进精密制造园、食品产业园、国家印刷包装产业基地等项目，布局实施商贸、物流、学校、医院等服务业项目。西区进一步提升集聚水平，提高土地利用率，电子专业市场、陆地港年内投入运行。井开区全年实现规模工业主营业务收入480亿元、增长20%。

　　壮大县域经济实力。完善县域经济发展政策措施和环境条件，推行差异化考核，促进各县市区做大总量、做强财力、做富百姓、做美城乡，形成一批产业集群。力争永丰县循环经济和碳酸钙产业达到百亿规模；吉安县数字视听、吉水县粮油食品2个产业达到80亿规模；泰和县触控显示、新干县盐化工、吉安县食品饮料、永新县皮制品、安福县液压机电、吉州区装备制造和通讯传输设备7个产业达到50亿规模。力争全市财政总收入过10亿元的县区达到10个。

　　（三）推进工业升级发展，加速新型工业化进程。深入实施工业化核心战略，围绕强攻工业，做规模上量级，做板块上能级，做集群上水平，决战规模工业主营业务收入3000亿元。

　　加快产业集聚。推进战略性新兴产业与传统产业融合发展，着力加快电子信息、生物医药、绿色食品、先进装备制造、新能源新材料等重点产业集聚提升，着力培育20个百亿产业集群。加快打造国家级电子信息产业基地，推进“产业高端、产品终端、科技尖端、服务前端”，重点发展触控显示、通讯终端、数字视听、半导体照明等6个百亿产业集群，主营业务收入突破500亿元，增长25%。生物医药产业重点打造生物药、中成药等产业集群，实现主营业务收入450亿元，增长24.5%。食品加工业重点推进井冈山卷烟厂异地改造完成主体工程，堆花酒业、燕京啤酒退城进园，赣泉啤酒靠大联强，以及茶叶深加工等项目，力争主营业务收入达到270亿元，增长26%。先进装备制造业重点打造数控机床、电力电缆、液压机电等产业集群，实现主营业务收入220亿元，增长25%。

　　推动科技协同创新。鼓励企业自主创新、集成创新和引进消化吸收再创新，更加注重加强与国内知名院校、科研院所合作，推进项目联合攻关、共建研发平台、组建产业技术创新联盟，加快建立企业为主体、产学研用协同创新体系。重点围绕电子信息、绿色食品、生物医药等产业，建设20个创新平台，开发30项省级新产品。推进质量体系建设，实施质量兴市战略。推动吉安高新产业园区升格国家高新区。注重人才引进和培养，建立激励机制，建设人才公寓，增强对人才的吸引力。

　　强化发展保障。积极推进工业园区扩区调区，深入开展园区闲置土地清理活动。完善园区配套，加快建设商贸中心、家庭公寓等生活服务设施，加快完善标准厂房、用水用电、仓储物流等生产配套条件，新增标准厂房100万平方米，建成公租房5000套。加强企业用工帮扶，用好劳动力资源普查成果，帮助企业与务工人员建立“点对点”招工通道。加快电网建设，开工建设8个110千伏输变电工程。

　　（四）全面推进服务业发展，拓展经济增长新空间。坚持生产性服务业与生活性服务业并重、新兴服务业与传统服务业并举，促进服务业发展提速、比重提高、水平提升，力争服务业实现增加值385亿元，增长11%。

　　做大做强旅游业。围绕打造全国红色旅游精品城市、建设旅游强市，深入实施“三山一江”战略，加快推进红色旅游一体化发展，推进国家旅游扶贫试验区建设，建立旅游资源开发运营利益共享机制，组建山上山下联动发展的旅游开发公司。打造精品景区，按照5A级景区标准，加快推进井冈山罗浮片区综合开发，以及青原山、武功山、渼陂、富田、吉州窑、永新三湾等重点景区建设。构建精品线路，放大产业联动效应。提供精品服务，培育大型旅行社，加快中心城区五星级宾馆和各县区四星级宾馆建设步伐。积极挖掘文化内涵和地域特色，推进农旅结合、红绿古结合，推动文旅互动、以文兴旅。

　　繁荣发展商贸业。加快中心城区市场体系建设，天虹商场、华润商城、铜锣湾商业慢行系统等项目竣工投入使用，儒林里特色古街、电子数码城等项目完成主体工程，新建、改造一批标准化菜市场。规划布局木材市场、花卉市场等专业市场。推进县城高端商贸综合体建设，改造低端商业街、饮食城，提升商业品质。引导乡镇规划建设高水平超市、农贸市场，推进商贸网点向农村和社区延伸。

　　加快发展现代物流业。继续完善“一中心、一平台、多园区”物流体系，建设新干河西、泰和南方、峡江现代、吉水城西、遂川东区、万安西港、永新城北等物流园，继续抓好市河西综合物流园等项目建设，建成永丰城北物流园。进一步拓展信息网络，提升井开区物流园综合物流和铁海联运功能，建立连接工业园区、各大市场和生产企业的信息系统，扩大作业规模。完善各物流园区运作机制，强化仓储配送、汽修汽配、住宿餐饮等综合服务功能，提升物流服务水平。

　　培育发展新兴产业。大力发展电子商务业，抓紧规划布局一批电子商务集聚区，引进建设配送中心、仓储中心、结算中心。引导传统商贸企业、生产企业应用电子商务，拓展网络市场。发展文化创意产业，推进动漫产业和衍生产品开发。推动书法、剪纸、木雕和农民画等地域文化产品的拓展提升。加快城南总部基地建设，尽早发挥经济效益。适应新的消费需求，鼓励和推进社会力量发展健康养老、信息消费、中介服务、教育培训等多种服务业态。

　　（五）积极推进结构调整，加快发展现代农业。优化产业结构，放活经营机制，大力发展设施农业、精致农业与高效农业,加快建设现代农业强市。

　　不断调优农业结构。推进粮食稳产增产，严格耕地保护，担当粮食安全责任，确保总产400万吨以上。在此基础上加快发展特色富民产业，大力实施“5150工程”，今年新增井冈蜜柚10万亩、绿色蔬菜2万亩、珍贵楠木10万亩、高产油茶8万亩、竹木花卉10万亩、茶叶3万亩、烟叶4万亩。保障主要农产品有效供给和价格基本稳定，力争生猪、肉牛、家禽总产增长4%—6%。积极创建绿色食品、有机食品基地，打造井冈绿色有机品牌。

　　放活农业经营机制。在稳定农户承包权的基础上，放活土地经营权，组建农村集体土地流转平台，新增流转35万亩，大力培育专业大户、家庭农场、龙头企业、农民合作社等新型经营主体，力争新增家庭农场200家、农民合作社500家。推动农产品精深加工，促进一产“接二连三”。推进井冈山农业科技园功能提升、滚动发展，建立井冈蜜柚研究基地。加速13个县市区农业科技示范园建设，加快提质扩园。

　　提高农业综合生产能力。加强水利建设，开工237座小（二）型病险水库除险加固工程、21条中小河流治理项目及重点圩堤除险加固工程，抓好渠系配套建设，力争新增和恢复灌溉面积10万亩，改善灌溉面积23万亩。新建高标准农田16万亩。做好中心城区禾河路堤结合项目前期工作。完善农技推广和基层水利服务体系，加强农业气象水文服务、动物防疫、植物病虫害防治、农产品质量检测体系建设，落实农机购置补贴政策，提高农业生产技术和安全保障能力。

　　（六）统筹推进城乡建设，不断提高城镇化水平。坚持城乡统筹、城乡同治，按照“大规划、大贯通、大组团、大盘活”要求，注重彰显“文化庐陵、山水吉安”特色魅力，做好“减量、透绿、留白、传承、坚守”五篇文章，加快以人为核心的城镇化进程，城镇化率提高2个百分点。

　　加强中心城区建设。以功能体系为牵引，形成大区域、多组团的整体推进格局。加强资金运作和项目实施，加快推进城南新区、赣江西堤、滨江新区、青原山对接区、高铁新区、井开区东区、吉安县城北新区，以及金樟组团开发建设。在中心城区实施198个城建重点项目，年内基本完成古后河绿廊二期、螺湖湾改造等64个项目，开工建设庐陵人文谷、庐陵现代艺术园等110个项目。进一步完善路、水、电、通讯网络，推动中心城区交通大贯通，加快形成“城市主干道+城市外环+绕城高速”三级城市交通网络。策应高铁建设，启动城西路网通达工程和高压电网布局调整。

　　完善城镇功能。加快县城新区拓展、老城改造步伐。加强公共设施建设，推进一批面向社区的公共文化设施建设。拓展生态宜居空间，推进县城公园向城中绿化通道和郊野公园延伸，每个县建成一个郊野公园。加快推进小城镇建设，完善公共服务设施，改善圩镇环境。推进产城互动，增强乡镇产业发展能力，进一步发展农业种养加和服务业，引导重点镇适度布局发展集约用地、生态环保的加工业项目。

　　建设美丽乡村。按照“五美”标准和“八不八多”要求，进一步扩建示范带、打造精品点，推进点线面结合，全面实施镇村联动，完成980个村点的美丽乡村建设。强化农民建房管控，健全从乡村规划、工匠培训、用地审批、规划执行、执法巡查到责任追究的一体化管控机制。突出卫生管理，推行“户分类、村收集、镇转运、县处理”为主的农村垃圾处理模式，建立农村环卫长效机制。

　　强化城镇管理和文化传承。加快户籍制度改革，积极推行居住证制度，推进农业转移人口市民化。启动"智慧城市"建设，整合现有信息化资源，推进数字化、网格化、精细化管理，提高城市管理和公共服务水平。深入推进“四城”同创，抓好中心城区全国文明城市、国家卫生城市、国家森林城市、省级园林城市创建工作。传承与坚守文化，最大限度地延续历史文脉、保留历史记忆。大力弘扬“崇文正气开放图强”的吉安城市精神，不断提高市民文明程度。

　　（七）深入推进全方位开放，提升开放型经济发展水平。坚持对外开放与对内开放并举，着力推进开放升级。

　　加大开放力度。大力实施全领域开放，力争引进内资480亿元，实际利用外资8亿美元。突出重点区域，巩固沿海招商，拓展境外招商，主招台商，努力打造江西台商集聚地。深入推进招大引强，加强与央企和科研院所的对接，紧盯世界500强、国内200强等知名企业，力争在引进优质、高端、领军型重大项目上取得突破。创新招商方式，推进以商招商、补链招商等多种模式。强化招商力量，坚持领导带头招商，加强以园区为基础的专业招商，积极探索和激励社会招商。

　　扩大外贸出口。大力引进和培育生产型出口企业，力争新增5家年出口超5000万美元的企业，超1亿美元的企业取得突破。进一步优化出口结构，大力实施科技兴贸战略，力争机电高新产品出口占比超过50%。积极推进贸易梯度转移重点承接地建设，推进井冈山出口加工区申报综合保税区，不断优化口岸平台。全市完成外贸出口37.2亿美元。

　　进一步提升开放的区位优势。支持配合跨区域重大项目建设，积极争取并大力支持昌吉赣客专、岳吉铁路、吉安绕城高速、新干-东乡高速、广昌-吉安高速、新干三湖航电枢纽等项目开工建设，支持推进江西成品油管道二期工程、西气东输三线工程等项目建设。加快市县重点项目建设，确保105国道中心城区改道、永和连心大桥在5月份以前建成通车，加快推进新井冈山大桥、庐陵大桥、井冈山机场扩建、亚行贷款吉安城市综合交通等项目建设。做好峡江水利枢纽库区移民安置后续工作，为年底6台机组并网发电做好蓄水准备。抓紧做好华能井冈山电厂三期前期准备，积极推进衡茶吉铁路东延、吉安至泉州铁路、井冈山至赣州铁路前期工作。

　　（八）加强生态环境建设，进一步增强绿色生态优势。着眼实现可持续发展、形成后发优势，大力推进生态建养、环境保护和节能减排，在全省生态文明示范区建设中主动作为、争当先锋。

　　加强生态建养。坚持生态立市，确立市立公园最严格的保护制度，在城镇区域设立“精品公园+生态绿廊+郊野公园”三级生态梯度，在广大乡村设立生态保护区，实行生态红线管控。科学设立吉泰走廊优强开发、重点开发、限制开发和禁止开发4个功能区，加快建设吉泰走廊百里生态廊道，中心城区新建4个郊野公园，走廊重点镇沿线规划建设一批生态示范区，形成走廊城镇组团的郊野生态绿色屏障。大力推进“森林城乡、绿色通道”建设，完成造林40万亩。

　　推进节能减排。严格落实节能减排目标责任制、“一票否决”制和区域限批制，全面完成节能减排目标任务。大力推进企业节能，促进能源资源循环利用、高效利用和清洁利用，确保全市万元工业增加值能耗下降4%以上。大力发展循环经济，积极创建国家循环经济示范城市、全国再生资源回收利用试点城市，抓好井开区和永丰工业园区省级循环化改造工作。

　　加大环境保护力度。开展净空、净水、净土行动。严把项目环保准入关，制定项目引进“负面清单”，从源头控制环境污染。严格落实项目环保“三同时”制度，加强环境评价跟踪管理。推进重点企业环境污染强制责任保险试点工作。加强重点污染源在线监测，加快环境空气监测预警体系建设，建成中心城区PM2.5监测系统。加强农村面源污染治理，重点推进畜禽清洁生产和农村清洁工程。大力实施水生态保护工程，开展集中式饮用水水源地污染整治，确保达标率保持100%。推进螺滩水库等应急备用水源地建设。加强水库水质监测、保护和治理，全面取缔施肥养殖，确保大中型水库全面达标，小型水库明显提升。

　　（九）加大民生保障力度，不断提升群众幸福指数。坚持尽力而为、量力而行和保基本、重托底、广覆盖的原则，抓好民生保障工作。

　　努力扩大就业。实施创业就业“123”工程，实现1000名返乡创业、2万名返乡务工、3万名就地务工就业。积极开展就业培训，完成工业园区定向培训3万人以上。落实税费减免、相关补贴、政府购买城乡公益性岗位等就业扶持政策，继续实施“三支一扶”计划。进一步创造就业机会，全市新增城镇就业5万人以上，转移农村劳动力7万人以上。

　　完善社会保障体系。加强社会保险扩面征缴，力争城乡居民养老保险参保率达到98%。建设70个城市社区居家养老点，300个以上农村老人幸福院。加快建立各种保障制度衔接和统一的社会救助体系，筑牢托底性社会保障制度。

　　推进保障性安居工程建设。新建城镇保障性住房1.53万套，基本建成6900套。突出抓好棚户区（城中村）改造，全市新开工各类棚户区改造1.93万户，其中中心城区8300户，实施城市非成套住房对象基本保障制度。加快无物业管理住宅小区改造，中心城区全面完成50户以上小区改造任务，各县市按照三年完成的要求年内共实施70个项目。完成农村危房年度改造任务。

　　加大扶贫攻坚力度。继续推进“六大扶贫工程”，更加突出产业扶贫和精准扶贫，实现8.5万贫困人口脱贫，完成移民搬迁1.2万人。深入实施农村水、电、路等基础设施“最后一公里三年攻坚计划”，完成农村公路建设2000公里。推进农村安全饮水工程，解决43万农村居民和9万农村师生安全饮水问题。

　　大力推进惠民工程。实施农村义务教育营养改善计划。开展圆梦助学活动，多渠道解决困难家庭子女就学问题。推进农村文化“三项活动”，继续巩固广播电视“村村通”和农家书屋建设。建立乡镇卫生院驻村巡诊制，消除卫生室空白村。开展新农合大病保险和市级统筹，建立农村医保“一卡通”。实施重大疾病免费救治，上海东方医院对先天性心脏病儿童实施免费手术。

　　（十）协调发展各项事业，促进社会全面进步。按照均衡化要求，推动教育、医疗、文化等公共服务资源向农村和社区延伸、提高，促进城乡社会事业协调发展。

　　坚持教育优先发展。推进吉安一中新校区、白鹭洲中学新校区二期、吉师附小改造等项目建设，确保今年秋季竣工投入使用。改善农村办学条件，实施1000个学校标准化建设项目。继续推进偏远农村中小学教学点布局建设，年内恢复73个教学点，招聘1000名以上农村教师。积极发展学前教育，合理布点建设幼儿园，加快推进闲置校舍改造幼儿园、村小增设附属幼儿园。大力发展职业教育，吉安职业技术学院实现秋季招生办学；推进职业教育与产业和企业对接，提高办学有效性。

　　繁荣文化体育事业。积极推进国家公共文化服务体系示范区、省级庐陵文化生态保护区等重点项目建设，完善文化服务网络。加强吉州窑遗址保护管理，推进白鹭洲书院修缮复原工程，完成市博物馆陈展并对外开放。抓好县级“两馆”维修改造、“三馆一站”免费开放和社区文化建设。加快建设市全民健身体育中心，推进社区健身场所建设，不断完善全民健身服务体系。

　　加快发展医疗卫生事业。扩大国家基本药物制度覆盖面，推进基层医疗卫生机构综合改革和公立医院改革。实施卫生能力提升工程，加快推进城南医院建设，开工建设市儿童医院，积极推进妇幼保健机构建设，加强乡镇卫生院医疗设备配备。加强人口计生工作，落实“单独两孩”政策，加大失独、独生子女伤残家庭帮扶力度。

　　加强和创新社会管理。推进社会治理社区化、单元化、网格化。推行“阳光信访”，畅通信访渠道，及时解决群众合理诉求。落实安全生产责任，提高企业本质安全水平，坚决遏制重特大安全事故发生。开展食品药品安全示范区创建活动，建立从生产、加工到流通、消费的全过程监管体系，保障人民群众饮食用药安全。推进国防和后备力量建设，加快军民融合深度发展。加强综合性应急管理，提高预防和处置突发事件能力。加大互联网监管力度，强化网络舆情引导和管理。深化平安建设，加快构建立体化社会治安防控体系，提升公众安全感和满意度，促进社会和谐稳定。

　　各位代表，完成2014年目标任务，全面深化改革，推进吉安发展升级、小康提速、绿色崛起、实干兴吉，对政府工作提出了新的更高要求。我们将以改革创新为引领，进一步加强政府自身建设，不断提升政府工作水平。我们将：大力解放思想。把解放思想贯穿于政府工作全过程，善于打破思维定式，跳出条条框框限制，推动各项工作与时俱进、创新发展。强化大局意识，着眼长远发展，敢于冲破利益藩篱，坚决革除体制机制的顽障痼疾。勇于突破狭隘视野，以更加宽广的眼界、更加开放的姿态，融入全省全国发展大局。强化务实操作。坚持以“啃硬骨头”的决心攻坚克难，“钉钉子”的精神狠抓落实，“锲而不舍、久久为功”的韧劲干出实效。建立健全工作部署、检查落实、监督反馈等工作制度，坚持重大项目领导挂点、重点工作现场办公。加强和改进政府工作绩效管理，充分调动政府工作人员开拓进取、勇于争先的积极性。密切联系群众。按照统一部署，认真开展群众路线教育实践活动，深入基层、深入一线，倾听群众呼声，了解群众愿望，多做暖民心、得民心的实事好事。畅通社情民意诉求渠道，认真做好群众来信来访工作，尽心尽力为群众排忧解难。规范政府行为。坚持依法行政，严格按照法定权限和程序行使职权、履行职责。自觉接受人大及其常委会的法律监督、工作监督和政协的民主监督，认真办理人大代表建议和政协提案。广泛听取各民主党派、工商联、无党派人士和人民团体的意见，主动接受社会监督和舆论监督，深入推进政务公开和政府信息公开，进一步完善新闻发布、社会公示和听证制度，保障人民群众的知情权、参与权、监督权。坚持从严治政。全面落实中央“八项规定”，坚决反对形式主义、官僚主义、享乐主义和奢靡之风，切实改进工作作风。加强政府工作人员教育管理，落实政府部门廉政建设责任制，建立健全惩治和预防腐败体系。加强对权力运行特别是行政审批、政府采购、土地出让、项目建设、公共资源交易等重点领域的行政监察和审计监督。推进公共资源网上交易。坚持厉行节约、勤俭办事，压缩“三公”经费，严禁铺张浪费，把有限的资金更多地用在加快发展、改善民生上，努力打造人民满意的廉洁政府。

　　各位代表！吉安正处于提速升级、进位赶超的关键时期。做好今年的经济社会发展工作任务艰巨、责任重大。让我们紧密团结在以习近平同志为总书记的党中央周围，在省委、省政府和市委的坚强领导下，进一步解放思想、改革创新，团结拼搏、锐意进取，奋力开创发展升级、小康提速、绿色崛起、实干兴吉的新局面，为建设开放繁荣秀美幸福新吉安、全面建成小康社会作出新的更大贡献！
